# Effects of adding sodium dichloroacetate to low-protein diets on nitrogen balance and amino acid metabolism in the portal-drained viscera and liver of pigs

**DOI:** 10.1186/s40104-020-00437-2

**Published:** 2020-04-13

**Authors:** Weizhong Sun, Yunxia Li, Zhiru Tang, Huiyuan Chen, Ke Wan, Rui An, Liuting Wu, Zhihong Sun

**Affiliations:** 1 Laboratory for Bio-feed and Molecular Nutrition, College of Animal Science and Technology, Southwest University, Chongqing, 400715 People’s Republic of China; 2 Institute of Animal Nutrition, Sichuan Agricultural University, Chengdu, 611130 People’s Republic of China

**Keywords:** Amino acid metabolism, Low-protein diet, Nitrogen excretion, Pig, Sodium dichloroacetate

## Abstract

**Background:**

Identifying regulatory measures to promote glucose oxidative metabolism while simultaneously reducing amino acid oxidative metabolism is one of the foremost challenges in formulating low-protein (LP) diets designed to reduce the excretion of nitrogen-containing substances known to be potential pollutants. In this study, we investigated the effects of adding sodium dichloroacetate (DCA) to a LP diet on nitrogen balance and amino acid metabolism in the portal-drained viscera (PDV) and liver of pigs.

To measure nitrogen balance, 18 barrows (40 ± 1.0 kg) were fed one of three diets (*n* = 6 per group): 18% crude protein (CP, control), 13.5% CP (LP), and 13.5% CP + 100 mg DCA/kg dry matter (LP-DCA). To measure amino acid metabolism in the PDV and liver, 15 barrows (40 ± 1.0 kg) were randomly assigned to one of the three diets (*n* = 5 per group). Four essential amino acids (Lys, Met, Thr, and Trp) were added to the LP diets such that these had amino acid levels comparable to those of the control diet.

**Results:**

The LP-DCA diet reduced nitrogen excretion in pigs relative to that of pigs fed the control diet (*P* < 0.05), without any negative effects on nitrogen retention (*P* > 0.05). There were no differences between the control and LP-DCA groups with respect to amino acid supply to the liver and extra-hepatic tissues in pigs (*P* > 0.05). The net release of ammonia into the portal vein and production rate of urea in the liver of pigs fed the LP-DCA diet was reduced relative to that of pigs fed the control and LP diets (*P* < 0.05).

**Conclusion:**

The results indicated that addition of DCA to a LP diet can efficiently reduce nitrogen excretion in pigs and maximize the supply of amino acids to the liver and extra-hepatic tissues.

## Background

The pork sector benefits the livelihoods of millions of producers and consumers but is also a source of serious nitrogen pollution. It is estimated that, worldwide, the pork sector releases 10 Tg of nitrogen into the environment annually, approximately 70% of which is lost to watercourses in the form of organic nitrogen and nitrates; the remainder is emitted into the atmosphere as nitrogenous gases (e.g., NH_3_, NO_X_, and N_2_O) [1]. In the past few decades, low-protein (LP) diets supplemented with indispensable amino acids have shown the potential to reduce nitrogen excretion [2–4], and numerous studies have shown no adverse effects on the growth performance of pigs when the dietary crude protein (CP) content was reduced by less than 2–3% [2, 5–7]. However, when the dietary protein content is reduced by ≥3%, it becomes necessary to add large amounts of amino acids to LP diets to meet animal requirements; otherwise pigs tend to show poor growth [8–13]. Nevertheless, it is deemed inadvisable to reduce the dietary CP content while simultaneously supplementing the diet with amino acids, as this increases both nitrogen intake and feed cost and reduces economic performance.

Urinary nitrogen typically accounts for 60–70% of total nitrogen excretion [14, 15], indicating that more attention should be paid to porcine urinary nitrogen. Theoretically, decreasing the entry of the main nitrogen precursors of urea into the liver is an important strategy for reducing urinary nitrogen. Ammonia (NH_3_), which is a direct nitrogen donor for urea, is mainly derived from the catabolism of amino acids, particularly those that represent a source of metabolic fuel and undergo extensive oxidative metabolism in the portal-drained viscera (PDV; including stomach, small and large intestines, pancreas, spleen, and omental fat) [16, 17]. Although glucose is a primary source of metabolic fuel that can be universally metabolized, large amounts of amino acids are metabolized in the PDV, for example, a large part of all non-essential amino acids (NEAAs) are oxidized by the absorptive epithelial cells (enterocytes) of mammalian small intestines [18–20]; 30–50% of essential amino acids (EAAs) in the diet may be catabolized by the small intestines in first-pass metabolism [21]. While glucose has little effect on the oxidative metabolism of amino acids, most amino acids, including glutamate and glutamine, can suppress glucose oxidation [22, 23]. Although intestinal mucosal amino acid metabolism is essential for maintaining intestinal integrity and function [24, 25], it should be noted that most amino acids metabolized in the intestines, particularly Gln, Glu, and Asp, are used as metabolic fuel [26] and produce nitrogen precursors for urea synthesis. Therefore, identifying the regulatory measures that promote glucose oxidation in the presence of amino acids is an important step for reducing nitrogen excretion and modifying LP diets.

With the aim of achieving this goal, we previously conducted a series of studies in which we made a number of key observations [17, 27, 28]. First, we found that NEAA insufficiency is a major disadvantage of the existing low-protein diets [17]. Second, we established that pyruvate, which is critical to energy metabolism [29, 30], could potentially replace the role of amino acids as a metabolic fuel and consequently reduce nitrogen excretion in pigs [27]. Third, we revealed that the pyruvate dehydrogenase kinase (PDK)/pyruvate dehydrogenase (PDH) axis is a key target for shifting amino acid oxidation to glucose/pyruvate metabolism [28]. In addition, we also found that culturing cells in the presence of sodium dichloroacetate (DCA) increased the phosphorylation of PDHA1/pyruvate dehydrogenase phosphatase 1 (PDP1), inhibited the phosphorylation of PDK1, and promoted increases in the mRNA and protein expression of glucose transporter 1 (GLUT1) and GLUT4 in cells, thereby increasing glucose consumption and reducing amino acid metabolism [28]. DCA, which is an inhibitor of PDK [31], binds to the N-terminal domain of PDK and promotes conformational changes, leading to the inactivation of kinase activity and reactivation of glucose oxidation [32]. DCA has been shown to reverse the Warburg effect of colorectal cancer [33–35], thereby reinstating oxidative phosphorylation. Oral administration has been shown to significantly increase in the activity of the PDH complex and tricarboxylic acid (TCA) cycle, not only in the platelets but also in various human tissues [36]. Previous studies have also indicated that DCA may be used as a metabolic modulator to enhance carbohydrate oxidative metabolism more precisely glucose oxidative metabolism, while reducing amino acid oxidative metabolism, thereby achieving the goal of reducing porcine nitrogen excretion. In addition, DCA is inexpensive, generally recognized as being safe [37–39], and has no detectable bone marrow, hepatic, renal, pulmonary, or cardiac toxicity [39]. Moreover, DCA-loaded mats had minimal effects on physical function after subcutaneous implantation or even attachment to the liver of mice [40].

We therefore hypothesized that DCA would be a suitable dietary additive to reduce nitrogen excretion in pigs by increasing amino acid utilization efficiency. Accordingly, the objective of the present study was to determine the effects of DCA supplementation of a LP diet (in which dietary CP was reduced by 4.5%) on nitrogen excretion and amino acid metabolism in the PDV and liver of pigs.

## Materials and methods

### Animal use and care

All animal procedures complied with the relevant ethical and animal welfare standards and were approved by the ethics committee of Southwest University.

Crossbred pigs (Yorkshire-Landrace sow × Duroc boar) were obtained from a local commercial swine farm and were reared individually in stainless-steel metabolic cages (1.8 m × 1.2 m × 0.75 m). All pigs had ad libitum access to fresh water. The room temperature was maintained at approximately 26 °C (range, 25.4–26.6 °C) using thermostatically controlled heaters and exhaust fans.

### Assessment of nitrogen balance

To measure nitrogen excretion, 18 barrows (40 ± 1.0 kg) were randomly assigned to receive one of three dietary treatments: 18% CP (control), 13.5% CP (LP), and 13.5% CP + 100 mg DCA/kg dry matter (LP-DCA). The control diet was formulated according to the recommendations of the National Research Council [41] and Lys, Met, Thr, and Trp were added to the LP diets (Table 1), making the actual amino acid intake (with the exception of Lys, Met, Thr, and Trp) of pigs in the LP and LP-DCA groups lower than that of pigs in the control group (Table 2). The dose of DCA selected for use in this study was based on that reported in the literature [38, 42]. The experimental period lasted 14 d, including habituation for the first 7 d and sample collection for the remainder of the period. The daily feed (45 g/kg body weight) was provided as two equal meals at 07:00 and 17:00 h. From d 8 to d 14, the feed was supplemented with 0.3% TiO_2_ (as a digesta marker). Feces and urine were collected at 08:00, 15:00, and 23:00 h from d 11 to d 14. The samples were collected according to previously described methods [17]. Briefly, the collected feces (approximately 0.2 kg per pig per collection) were combined with 10% sulfuric acid (10 mL/100 g feces) and frozen at − 20 °C. All urine samples were collected in plastic bottles, weighed, recorded, and stored at − 20 °C. Five milliliters of 6 mol/L HCl was added daily to each urine collection bottle, thereby preventing evaporation and nitrogen loss. Feed intake was recorded daily during the sample collection period.

**Table 1:** Ingredients and composition of diets differing in crude protein content (dry matter basis, %)

Items	Diets	
	Control	LP
Ingredients		
Corn	61.20	74.55
Soybean meal	25.90	12.07
Wheat bran	7.80	7.39
Soybean oil	2.00	2.00
Lys	0.18	0.55
Met	0.05	0.12
Thr	0.01	0.24
Trp	0.00	0.08
Dicalcium phosphate	0.69	0.90
Calcium carbonate	0.87	0.80
Salt	0.30	0.30
1% Premix^a^	1.00	1.00
Total	100.00	100.00
Composition		
Metabolic energy, MJ/kg^b^	13.9	13.9
Crude protein^c^	18.0	13.5
Lys^c^	1.11	1.12
Met^c^	0.35	0.35
Thr^c^	0.73	0.73
Trp^c^	0.22	0.22
Arg^c^	1.01	0.76
His^c^	0.44	0.33
Ile^c^	0.71	0.55
Leu^c^	1.35	1.02
Phe^c^	0.79	0.61
Val^c^	0.68	0.52
Pro^c^	0.73	0.56
Asx (Asp+Asn)^c^	1.76	1.34
Ser^c^	0.74	0.57
Glx (Glu + Gln)^c^	2.95	2.23
Gly^c^	0.58	0.44
Ala^c^	0.79	0.60
Cys^c^	0.23	0.18
Tyr^c^	0.37	0.28
Calcium^d^	0.63	0.63
Phosphorus^d^	0.54	0.54

Abbreviations: *Control* 18.0% CP, *LP* 13.5% CP

^a^Providing the following per kg diet: Cu (as copper sulfate), 100 mg; Fe (as ferrous sulfate), 100 mg; Zn (as zinc oxide), 120 mg; Mn (as manganese sulfate), 20 mg; I (as calcium iodate), 0.3 mg; and Se (as sodium selenite), 0.3 mg; vitamin A, 3, 800 IU; vitamin D_3_, 800 IU; vitamin E, 10 IU; vitamin K, 1 mg; choline, 200 mg; pantothenic, 5 mg; vitamin B_2_, 2 mg; folic acid, 0.8 mg; niacin, 10 mg; vitamin B_1_, 1 mg; vitamin B_6_, 1 mg; biotin, 0.08 mg; vitamin B12, 0.01 mg

^b^Calculated values

^c^Values for standardized ileal digestible concentrations of amino acids in diets were calculated using standardized ileal digestible coefficients for the various ingredients provided by NRC (2012) [41]

^d^Analyzed values

**Table 2:** The effects of adding sodium dichloroacetate (DCA) to low-protein diets on the dry matter and amino acid intakes of 40-kg pigs in a nitrogen balance experiment

Items	Treatments			SEM	*P*
	Control	LP	LP-DCA		
Intake of feed, kg	2.12	2.20	2.18	0.04	0.332
Intakes of amino acids, g/d					
Lys	24.9	24.0	23.4	0.18	0.417
Met	7.40	7.12	6.97	0.05	0.514
Thr	16.7	16.2	15.8	0.12	0.889
Trp	4.43	4.29	4.19	0.04	0.356
Arg	21.9^a^	15.8^b^	15.3^b^	0.13	<.001
His	9.63^a^	7.14^b^	6.99^b^	0.06	<.001
Ile	15.3^a^	11.4^b^	11.3^b^	0.10	<.001
Leu	29.3^a^	21.5^b^	21.1^b^	0.17	<.001
Phe	17.1^a^	12.8^b^	12.6^b^	0.11	<.001
Val	14.8^a^	10.8^b^	10.7^b^	0.10	<.001
Pro	15.9^a^	11.7^b^	11.5^b^	0.10	<.001
Asx (Asp+Asn)	38.2^a^	28.5^b^	28.1^b^	0.23	<.001
Ser	16.0^a^	11.9^b^	11.8^b^	0.10	<.001
Glx (Glu + Gln)	64.0^a^	47.7^b^	47.1^b^	0.39	<.001
Gly	12.6^a^	9.26^b^	9.08^b^	0.07	<.001
Ala	17.1^a^	12.3^b^	12.2^b^	0.11	<.001
Cys	9.38^a^	6.90^b^	6.93^b^	0.06	<.001
Tyr	8.03^a^	6.06^b^	5.93^b^	0.05	<.001
EAAs	162^a^	131^b^	128^b^	1.04	<.001
NEAAs	181^a^	134^b^	133^b^	1.10	<.001
TAAs	343^a^	265^b^	261^b^	2.13	<.001

Data are presented as means (*n* = 6)

Abbreviations: *Control* 18.0% CP, *CP* Crude protein, *DM* Dry matter, *EAAs* Essential amino acids, *NEAAs* Non-essential amino acids, *LP* 13.5% CP, *LP-DCA* 13.5% CP + 100 mg DCA/kg DM, *SEM* Standard error of mean, *TAAs* Total amino acids

^a,b^Values within a row with different superscripts differ significantly (*P* < 0.05)

At the end of the sample collection period, the urine and feces samples obtained from each pig were pooled per pig, mixed, and divided into subsamples. The fecal subsamples were dried, ground, and passed through a 1-mm screen for nutrient analysis, with all samples being analyzed in duplicate. The dry matter and organic matter content in the diet and feces; the CP content in the diet, urine, and feces; the amounts of phosphorus and calcium in the diet; and the amounts of titanium in the feces and diet were analyzed according to previously described methods [43, 44]. The energy in the feed, feces, and urine was determined using an adiabatic bomb calorimeter (C5000; IKA Werke GmbH, Staufen im Breisgau, Germany). The energy content of urine was determined after freeze-drying approximately 50 mL of urine in polyethylene bags. After hydrolysis with 6 mol/L at 110 °C, the amounts of amino acids (with the exception of Trp, Cys, and Met) in the feed were measured using an automatic amino acid analyzer (L-8900; Hitachi, Tokyo, Japan). The analytical procedures used for Trp, Cys, and Met followed previously described methods [45].

### Assessment of nutrient fluxes

After fasting for 24 h, 15 barrows (40 ± 1.0 kg) were surgically catheterized in the carotid artery, hepatic vein, portal vein, and mesenteric vein using polyurethane (2.41 mm OD) under isoflurane anesthesia and sterile conditions according to previously described methods [46]. After surgery, pigs were maintained in metabolism cages for recovery and received an intramuscular injection of penicillin twice daily (1.6 × 10^7^ units) for 7 d. Catheters were flushed daily aseptically with 200 IU of heparinized normal saline to maintain their patency and were protected in pockets attached to a jacket made of size 7 Surgilast (Glenwood Laboratories Canada, Oakville, ON, Canada). Wound Clear Spray (Guangan Animal Health Co., Changsha, Hunan, China) was applied to the sutures and to the exit site of catheters.

One week after surgery (or at the point at which pigs achieved > 80% of their pre-surgery feed intake for than 2 d), the pigs were randomly assigned to one of the three dietary treatments: 18% CP (control), 13.5% CP (LP), or 13.5% CP + 100 mg DCA/kg dry matter (LP-DCA). The experiment continued for 7 d, with blood samples collected on the final day. During the experimental period, the feed (45 g/kg body weight) was divided into three equal portions, provided at 08:00, 14:00, and 20:00 h. On the day of blood sampling, the animals received a priming dose of *p*-aminohippuric acid (PAH) solution (0.75 mL/kg; 0.14 μmol/mL) via the mesenteric vein at 07:30 h, followed by a constant infusion of PAH solution at a rate of 3.2 mL/kg/h. At 08:30, 10:00, 12:00, 14:30, and 17:30 h, 5 mL blood samples were collected from the carotid artery, portal vein, and hepatic vein, to which sodium heparin solution (100 IU/mL) was added as an anticoagulant. The blood samples were immediately placed on ice and centrifuged at 3,000×*g* for 10 min at 4 °C, and the plasma was stored at − 20 °C. The plasma obtained from each site at the five time points was pooled for each pig, and the concentration of PAH in the plasma was measured following previously described methods [47] with some modification [48]. The hepatic vein plasma flow (HVPF; mL/kg/h) and the portal vein plasma flow (PVPF; mL/kg/h) were calculated using the following equations [17, 49]:

$$ \mathrm{HVPF}={C}_{\mathrm{i}}\times \mathrm{IR}/\left({\mathrm{PAH}}_{\mathrm{hv}}-{\mathrm{PAH}}_{\mathrm{ca}}\right) $$

$$ \mathrm{PVPF}={C}_{\mathrm{i}}\times \mathrm{IR}/\left({\mathrm{PAH}}_{\mathrm{pv}}-{\mathrm{PAH}}_{\mathrm{ca}}\right) $$

where *C*_i_ is the concentration of infused PAH solution (mg/mL); IR is the infusion rate (mL/kg/h) of PAH; and PAH_hv_, PAH_ca_, and PAH_pv_ are the PAH concentrations (mg/mL) in the hepatic vein, carotid artery, and portal vein, respectively. The hepatic artery plasma flow (HAPF; mL/kg/h) was then calculated as follows:

$$ \mathrm{HAPF}=\mathrm{HVPF}-\mathrm{PVPF} $$

The concentrations of plasma urea and NH_3_ were measured following previously described procedures [50]. The analysis of fresh plasma NH_3_ was performed within 2 h of blood collection. The frozen plasma samples were thawed at 4 °C and precipitation of the proteins therein was carried out as follows. Briefly, 1 mL of the sample and 2.5 mL of 7.5% (w/v) trichloroacetic acid solution were mixed thoroughly and centrifuged at 12,000×*g* and 4 °C for 15 min. The supernatant was collected and analyzed for amino acids using an automatic amino acid analyzer (L-8900; Hitachi, Tokyo, Japan). The fluxes of plasma amino acids (mg/kg/h), NH_3_ (μmol/kg/h), and urea (mmol/kg/h) across the carotid artery, portal vein, or hepatic vein were calculated as the concentration of a plasma nitrogen-containing compound in a vein multiplied by the plasma flow across the vein. The PDV balance of a nitrogen-containing compound was calculated as follows:

$$ \mathrm{PVPF}\times \left(\mathrm{the}\ \mathrm{concentration}\ \mathrm{of}\ \mathrm{plasma}\ \mathrm{nitrogen}-\mathrm{containing}\ \mathrm{compound}\ \mathrm{in}\ \mathrm{the}\ \mathrm{portal}\ \mathrm{vein}-\mathrm{the}\ \mathrm{concentration}\ \mathrm{of}\ \mathrm{the}\ \mathrm{corresponding}\ \mathrm{compound}\ \mathrm{in}\ \mathrm{the}\ \mathrm{carotid}\ \mathrm{artery}\right). $$

A positive value means net uptake and a negative value signifies net release. The hepatic balance of nitrogen-containing compound was calculated as follows:

the concentration of nitrogen-containing compound in the hepatic artery × HAPF + the AA concentration in the portal vein × PVPF – the AA concentration in the hepatic vein × HVPF. A positive value indicates that the amount of nitrogen-containing compound leaving the liver is smaller than that entering the liver, whereas a negative value indicates that the amount of nitrogen-containing compound leaving the liver is greater than that entering the liver.

### Statistical analysis

Data were analyzed based on a randomized complete block design considering the pig as the experimental unit. The MIXED procedure of SAS (SAS Institute, Cary, NC, USA) was used to analyze the variables. The fixed effect consisted of dietary treatment (control, LP, and LP-DCA), with the pig as the random component. The model used was as follows:

$$ {\mathrm{Y}}_{\mathrm{i}\mathrm{jk}}=\upmu +{\mathrm{T}}_{\mathrm{i}}+{\mathrm{L}}_{\mathrm{i}}+{\mathrm{A}}_{\mathrm{j}}+{\mathrm{E}}_{\mathrm{i}\mathrm{jk}} $$

where Y_ijk_ is the dependent variable, μ is the overall mean, T_i_ is the dietary treatment, L_i_ is the period effect, A_i_ is the pig effect, and E_ijk_ is the residual error. Differences between treatment means were determined by Tukey multiple comparison tests. Results are reported as means ± SEM, and *P* < 0.05 was considered statistically significant.

## Results

### Nitrogen balance

As shown in Table 3, LP diets (LP and LP-DCA) reduced urinary nitrogen excretion, fecal nitrogen excretion, and total nitrogen excretion in pigs (*P* < 0.05). Compared with the control and LP-DCA diets, the LP diet reduced the retention of nitrogen in pigs (*P* < 0.05). Furthermore, we found that the digestive energy digestibility and metabolic energy/digestive energy ratio of pigs in the LP-DCA group were both higher than those of pigs in the control group (*P* < 0.05).

**Table 3:** The effects of adding sodium dichloroacetate (DCA) to low-protein diets on the nitrogen balance and apparent digestibility of other nutrients in 40-kg pigs

Items	Treatments			SEM	*P*
	Control	LP	LP-DCA		
Nitrogen utilization					
Average daily feed intake	1.60	1.66	1.66	0.07	0.543
Intake of nitrogen, g/d	46.0^a^	35.8^b^	35.9^b^	0.54	<.001
Urinary nitrogen, g/d	13.6^a^	10.4^b^	7.52^c^	0.17	<.001
Fecal nitrogen, g/d	9.03^a^	5.87^b^	5.54^b^	0.11	<.001
Total nitrogen excretion, g/d	22.6^a^	16.3^b^	13.1^c^	0.26	<.001
Retention of nitrogen, g/d	23.4^a^	19.5^b^	22.8^a^	0.35	<.001
Urinary nitrogen/intake of nitrogen, %	29.6^a^	29.1^a^	20.9^b^	0.28	<.001
Fecal nitrogen/intake of nitrogen, %	19.6^a^	16.4^b^	15.4^b^	0.20	<.001
Total nitrogen excretion/intake of nitrogen, %	49.2^a^	45.4^b^	36.4^c^	0.36	<.001
Urinary nitrogen/total nitrogen excretion, %	60.2^ab^	63.9^a^	57.6^b^	0.38	<.001
Fecal nitrogen/total nitrogen excretion, %	39.9^b^	36.1^c^	42.4^a^	0.38	<.001
Retention of nitrogen/intake of nitrogen, %	50.8^c^	54.6^b^	63.6^a^	0.36	<.001
Other nutrient utilization, %					
DM digestibility	75.3^b^	77.1^ab^	79.5^a^	0.48	<.001
Organic matter digestibility	74.9^b^	77.9^ab^	79.4^a^	0.40	<.001
Digestive energy digestibility	70.8^b^	73.4^ab^	75.8^a^	0.57	<.001
Metabolic energy/digestive energy ratio	84.1^b^	87.2^ab^	90.2^a^	0.44	<.001

Data are presented as means (*n* = 6)

Abbreviations: *Control*, 18.0% CP, *CP* Crude protein, *DM* Dry matter, *LP* 13.5% CP, *LP-DCA* 13.5% CP + 100 mg DCA/kg DM, *SEM* Standard error of mean

^a,b,c^ Values within a row with different superscripts differ significantly (*P* < 0.05)

### AA metabolism in the PDV and liver

As shown in Table 4, with the exception of Lys, Trp, and urea, the concentrations of plasma nitrogenous compounds in the portal vein of pigs fed the LP diet were lower than those of pigs fed the control diet (*P* < 0.05). The concentrations of plasma Met, Arg, His, Ile, Phe, Val, Pro, Ser, Gly, Ala, and EAAs in the hepatic vein of pigs fed the LP were lower than those of pigs fed the control diet (*P* < 0.05). Similarly, the concentrations of plasma Gly, Ala, and NH_3_ in the portal vein and those of plasma Gly and Ala in the hepatic vein of pigs fed the LP-DCA diet were lower than those of pigs fed the control diet (*P* < 0.05). Furthermore, the concentrations of plasma Thr, Pro, Ser, and Gln in the portal vein and those of plasma Met, Arg, His, Ile, Val, Pro, and EAAs in the hepatic vein of pigs fed the LP diet were lower than those of pigs fed the LP-DCA diet (*P* < 0.05).

**Table 4:** The effects of adding sodium dichloroacetate (DCA) to low-protein diets on the concentrations of amino acids (mg/dL), NH_3_ (μmol/L), and urea (mmol/L) in the portal vein, hepatic artery, and hepatic vein of 40-kg pigs

Items	Portal vein			SEM	*P*	Hepatic artery			SEM	*P*	Hepatic vein			SEM	*P*
	Control	LP	LP-DCA			Control	LP	LP-DCA			Control	LP	LP-DCA		
Lys	3.30	2.93	3.15	0.07	0.076	2.42	2.14	2.32	0.06	0.071	2.88	2.57	2.87	0.08	0.058
Met	1.39^a^	1.18^b^	1.32^ab^	0.03	0.023	1.00^a^	0.86^b^	0.96^ab^	0.03	0.049	1.22^a^	1.03^b^	1.20^a^	0.03	0.012
Thr	3.97^a^	3.55^b^	3.86^a^	0.09	0.028	3.22	2.91	3.15	0.09	0.100	3.65	3.25	3.63	0.11	0.054
Trp	0.64	0.56	0.59	0.01	0.068	0.45	0.40	0.44	0.01	0.077	0.50	0.46	0.51	0.01	0.053
Arg	2.95^a^	2.56^b^	2.80^ab^	0.06	0.024	2.21	1.98	2.11	0.06	0.130	2.55^a^	2.21^b^	2.53^a^	0.06	0.026
His	2.08^a^	1.74^b^	1.94^ab^	0.04	0.014	1.63	1.43	1.51	0.04	0.087	1.83^a^	1.53^b^	1.78^a^	0.04	0.009
Ile	2.32^a^	1.97^b^	2.16^ab^	0.08	0.026	1.51^a^	1.30^b^	1.40^ab^	0.04	0.038	1.98^a^	1.69^b^	1.93^a^	0.05	0.013
Leu	3.48^a^	3.07^b^	3.29^ab^	0.07	0.024	2.50	2.26	2.41	0.07	0.157	3.12	2.79	3.05	0.08	0.057
Phe	2.77^a^	2.37^b^	2.60^ab^	0.06	0.018	1.99	1.78	1.89	0.05	0.136	2.45^a^	2.11^b^	2.38^ab^	0.06	0.030
Val	2.22^a^	1.93^b^	2.12^ab^	0.06	0.024	1.86	1.64	1.79	0.05	0.092	2.04^a^	1.78^b^	2.00^a^	0.05	0.040
Pro	1.86^a^	1.54^b^	1.78^a^	0.04	0.002	1.44	1.28	1.41	0.04	0.143	1.60^a^	1.37^b^	1.59^a^	0.04	0.017
Asn	3.30^a^	2.94^b^	3.16^ab^	0.07	0.046	2.81	2.55	2.72	0.07	0.167	3.06	2.78	2.97	0.07	0.116
Ser	2.15^a^	1.66^b^	2.01^a^	0.04	<.001	1.86^a^	1.53^b^	1.78^a^	0.05	0.004	2.04^a^	1.72^b^	1.94^ab^	0.05	0.019
Gln	4.52^a^	3.94^b^	4.35^a^	0.09	0.008	4.87	4.57	4.77	0.13	0.429	5.32	5.10	5.25	0.13	0.780
Gly	5.56^a^	4.95^b^	4.57^b^	0.11	<.001	3.35	3.22	3.25	0.09	0.660	4.39^a^	3.98^b^	3.93^b^	0.10	0.018
Ala	5.88^a^	5.10^b^	4.69^b^	0.11	<.001	3.50	3.32	3.30	0.09	0.300	4.59^a^	4.07^b^	3.92^b^	0.11	0.007
Cys	1.94^a^	1.66^b^	1.82^ab^	0.06	0.016	1.63^a^	1.39^b^	1.52^ab^	0.04	0.016	1.84	1.63	1.79	0.04	0.219
Tyr	2.29^a^	1.98^b^	2.18^ab^	0.07	0.016	2.03	1.82	1.95	0.05	0.173	2.13	1.90	2.10	0.05	0.175
EAAs	25.1^a^	21.9^b^	24.1^ab^	0.63	0.017	18.8	16.7	18.0	0.49	0.103	22.3^a^	19.5^b^	21.9^a^	0.54	0.037
NEAAs	27.5^a^	23.6^b^	24.9^ab^	0.66	0.013	21.5	19.7	20.7	0.57	0.257	25.0	22.6	23.5	0.59	0.103
TAAs	52.6^a^	45.5^b^	49.0^ab^	1.19	0.018	40.3	36.35	38.7	1.06	0.166	47.2	42.0	45.4	1.13	0.075
NH_3_	93.7^a^	80.2^b^	69.7^c^	2.42	<.001	44.5	45.0	41.4	1.26	0.292	49.3	48.0	44.0	1.38	0.083
Urea	7.70	7.68	7.12	0.17	0.722	7.93	7.90	6.91	0.22	0.310	8.76	8.48	7.63	0.21	0.163

Data are presented as means (*n* = 5)

Abbreviations: *CP* Crude protein, *DM* Dry matter, *EAAs* Essential amino acids, *NEAAs* Non-essential amino acids, *LP* 13.5% CP, *LP-DCA* 13.5% CP + 100 mg DCA/kg DM, *SEM* Standard error of mean, *TAAs* Total amino acids

^a,b,c^ Values within a row with different superscripts differ significantly (*P* < 0.05)

As shown in Table 5, plasma flow across the hepatic artery of pigs fed the LP diet was lower than that of pigs in the control group (*P* < 0.05). However, there were no significant differences in plasma flow across the portal and hepatic veins in pigs among the three groups (*P* > 0.05). The fluxes of plasma nitrogenous compounds across the portal vein (with the exception of Lys, Asn, and urea) and the fluxes of plasma nitrogen-compounds across the hepatic vein (with the exception of Asn, Gln, Tyr, NH_3_, and urea) were lower in pigs fed the LP diet than those of pigs fed the control diet (*P* < 0.05). The fluxes of plasma Gly, Ala, and NH_3_ across the portal vein and the fluxes of plasma Gly, Ala, and urea across the hepatic vein of pigs fed the LP-DCA diet were lower than those of pigs fed the control diet (*P* < 0.05). Furthermore, the fluxes of plasma Met, Thr, Arg, His, Pro, Ser, Gln, and Tyr across the portal vein and the fluxes of plasma nitrogenous compounds across the hepatic vein of pigs fed the LP diet (with the exception of Leu, Asn, Gln, Gly, Ala, Cys, NEAAs, NH_3_, and urea) were lower than those of pigs fed the LP-DCA diet (*P* < 0.05).

**Table 5:** The effects of adding sodium dichloroacetate (DCA) to low-protein diets on the fluxes of plasma (mL/kg/h), amino acids (mg/kg/h), NH_3_ (μmol/kg/h), and urea (mmol/kg/h) across the portal vein, hepatic artery, and hepatic vein of 40-kg pigs

Items	Portal vein			SEM	*P*	Hepatic artery			SEM	*P*	Hepatic vein			SEM	*P*
	Control	LP	LP-DCA			Control	LP	LP-DCA			Control	LP	LP-DCA		
Plasma	1614	1627	1659	35.6	0.878	409^a^	346^b^	404^a^	13.9	0.004	2023	1973	2063	51.3	0.330
Lys	53.4	47.5	52.1	1.25	0.060	9.88^a^	7.59^b^	9.36^a^	0.23	<.001	58.4^a^	50.8^b^	58.9^a^	2.58	0.028
Met	22.5^a^	19.1^b^	21.7^a^	0.53	0.024	4.08^a^	3.05^b^	3.86^a^	0.09	<.001	24.6^a^	20.5^b^	24.6^a^	0.99	<.001
Thr	64.5^a^	57.5^b^	63.9^a^	1.52	0.037	13.2^a^	10.3^b^	12.7^a^	0.32	<.001	73.8^a^	64.4^b^	74.6^a^	3.21	0.030
Trp	10.4^a^	9.02^b^	9.84^ab^	0.23	0.045	1.84^a^	1.42^b^	1.76^a^	0.05	<.001	10.2^a^	9.03^b^	10.3^a^	0.48	0.040
Arg	47.6^a^	41.5^b^	46.3^a^	1.10	0.023	9.03^a^	7.02^b^	8.54^a^	0.23	<.001	51.6^a^	43.8^b^	51.9^a^	2.42	0.020
His	33.6^a^	28.3^b^	32.0^a^	0.77	0.007	6.60^a^	5.07^b^	6.11^a^	0.17	<.001	37.0^a^	30.3^b^	36.6^a^	1.76	<.001
Ile	37.5^a^	31.9^b^	35.7^ab^	0.86	0.013	6.16^a^	4.62^b^	5.64^a^	0.18	<.001	40.0^a^	33.5^b^	39.6^a^	1.70	<.001
Leu	56.3^a^	49.9^b^	54.5^ab^	1.29	0.040	10.2^a^	8.01^b^	9.73^a^	0.26	<.001	63.3^a^	55.1^b^	62.6^ab^	2.72	0.047
Phe	44.7^a^	38.4^b^	42.7^ab^	1.02	0.014	8.15^a^	6.32^b^	7.63^a^	0.20	<.001	49.6^a^	41.8^b^	48.9^a^	2.10	0.021
Val	35.9^a^	31.4^b^	35.1^ab^	0.84	0.040	7.60^a^	5.83^b^	7.24^a^	0.18	<.001	41.3^a^	35.2^b^	41.1^a^	2.99	0.031
Pro	30.1^a^	25.0^b^	29.5^a^	0.69	0.004	5.90^a^	4.55^b^	5.69^a^	0.14	<.001	32.5^a^	27.1^b^	32.7^a^	2.09	<.001
Asn	53.3	47.6	52.3	1.25	0.068	11.5^a^	9.03^b^	11.0^a^	0.27	<.001	61.9	55.0	61.0	0.96	0.087
Ser	34.7^a^	26.9^b^	33.2^a^	0.80	<.001	7.61^a^	5.42^b^	7.17^a^	0.17	<.001	41.3^a^	34.1^b^	39.9^a^	1.65	0.015
Gln	73.0^a^	63.9^b^	71.9^a^	1.70	0.012	19.9^a^	16.2^b^	19.3^a^	0.48	<.001	108	101	108	4.62	0.707
Gly	89.8^a^	80.2^b^	75.6^b^	1.97	0.001	13.8^a^	11.4^b^	13.1^ab^	0.38	0.018	89.0^a^	78.7^b^	80.6^b^	3.80	0.021
Ala	95.0^a^	82.6^b^	77.7^b^	2.07	<.001	14.3^a^	11.8^b^	13.3^ab^	0.42	0.028	92.8^a^	80.5^b^	80.9^b^	3.92	0.008
Cys	31.3^a^	26.8^b^	30.1^ab^	0.72	0.027	6.71^a^	4.92^b^	6.14^b^	0.17	<.001	36.8^a^	32.3^b^	35.9^ab^	1.52	0.025
Tyr	37.1^a^	32.2^b^	36.2^a^	0.86	0.028	8.26^a^	6.45^b^	7.87^a^	0.18	<.001	43.1	37.7	43.2	1.94	0.150
EAAs	406^a^	354^b^	393^ab^	9.39	0.029	76.8^a^	59.3^b^	72.6^a^	1.92	<.001	450^a^	384^b^	449^a^	22.5	0.023
NEAAs	444^a^	383^b^	408^ab^	10.0	0.019	88.0^a^	69.8^b^	83.5^a^	2.58	<.001	505^a^	446^b^	482^ab^	17.0	0.046
TAAs	850^a^	737^b^	801^ab^	19.4	0.018	165^a^	129^b^	156^a^	3.93	<.001	955^a^	831^b^	931^a^	36.5	<.001
NH_3_	151^a^	130^b^	113^c^	3.23	<.001	18.4^a^	18.6^a^	16.7^b^	0.54	0.034	99.9	97.4	89.3	2.74	0.098
Urea	124	125	117	2.94	0.908	33.4^a^	27.3^b^	27.9^b^	0.84	0.015	177^a^	167^ab^	157^b^	5.53	0.021

Data are presented as means (*n* = 5)

Abbreviations: *Control* 18.0% CP, *CP* Crude protein, *DM* Dry matter, *EAAs* Essential amino acids, *NEAAs* Non-essential amino acids, *LP* 13.5% CP, *LP-DCA* 13.5% CP + 100 mg DCA/kg DM, *SEM* Standard error of mean, *TAAs* Total amino acids

^a,b,c^ Values within a row with different superscripts differ significantly (*P* < 0.05)

Compared with the control diet, the LP diet reduced the net release of all nitrogenous compounds (with exception of Lys and Cys) into the portal vein (*P* < 0.05); the LP-DCA diet reduced the net release of Trp, Gly, Ala, NEAAs, total amino acids (TAAs), and NH_3_ into the portal vein (*P* < 0.05) (Table 6). Moreover, we observed that the net release of Met, Thr, Arg, His, Ile, Leu, Phe, Val, Pro, Asn, Ser, Gln, Tyr, EAAs, TAAs, and NH_3_ to the portal vein of pigs fed the LP-DCA diet was higher than that of pigs fed the LP (*P* < 0.05). The net release of Gly, Ala, and NH_3_ to the portal vein of pigs fed the LP-DCA diet was lower than that of pigs fed the LP diet (*P* < 0.05).

**Table 6:** The effects of adding sodium dichloroacetate (DCA) to low-protein diets on the portal-drained viscera balance of amino acids (mg/kg/h) and NH_3_ (μmol/kg/h) in pigs^*^

Items	Treatments			SEM	*P*
	Control	LP	LP-DCA		
Lys	14.3	12.9	14.2	0.29	0.087
Met	6.36^a^	5.16^b^	6.16^a^	0.12	0.005
Thr	12.2^a^	10.4^b^	12.3^a^	0.32	0.013
Trp	3.12^a^	2.56^b^	2.70^b^	0.05	0.031
Arg	11.9^a^	9.44^b^	11.8^a^	0.25	<.001
His	7.47^a^	5.06^b^	7.31^a^	0.17	<.001
Ile	13.2^a^	10.8^b^	13.0^a^	0.25	<.001
Leu	15.9^a^	13.2^b^	15.1^a^	0.31	0.001
Phe	12.5^a^	9.57^b^	11.9^a^	0.24	<.001
Val	5.83^a^	4.70^b^	5.78^a^	0.18	0.049
Pro	6.72^a^	4.20^b^	6.42^a^	0.15	<.001
Asn	7.80^a^	6.37^b^	7.79^a^	0.27	0.049
Ser	4.65^a^	2.19^b^	4.14^a^	0.18	<.001
Gln	-5.67^a^	−10.3^b^	−6.19^a^	0.69	0.002
Gly	35.4^a^	28.2^b^	22.6^c^	0.57	<.001
Ala	38.4^a^	28.8^b^	23.8^c^	0.60	<.001
Cys	4.74	4.20	4.86	0.15	0.176
Tyr	4.41^a^	2.64^b^	4.24^a^	0.20	0.001
EAAs	103^a^	83.8^b^	100^a^	2.13	<.001
NEAAs	96.5^a^	66.3^b^	67.7^b^	2.10	<.001
TAAs	199^a^	150^c^	168^b^	4.20	<.001
NH_3_	79.5^a^	56.8^b^	45.7^c^	1.46	<.001

Data are presented as means (*n* = 5)

Abbreviations: *Control* 18.0% CP, *CP* Crude protein, *DM* Dry matter, *EAAs* Essential amino acids, *NEAsA* Non-essential amino acids, *LP* 13.5% CP, *LP-DCA* 13.5% CP + 100 mg DCA/kg DM, *SEM* Standard error of mean, *TAAs* Total amino acids

^*^A positive value means the amount of nitrogen-containing compound entering the portal vein is greater than that entering the portal-drained viscera through the artery; a negative value means the amount of nitrogen-containing compound entering the portal vein is less than that entering the portal-drained viscera through the artery

^a,b,c^Values within a row with different superscripts differ significantly (*P* < 0.05)

Compared with pigs fed the control diet, those fed the LP diet showed reduced metabolism of Met, Trp, Ile, Pro, Asn, Se, Gln, Gly, Ala, Cys, Tyr, EAAs, NEAAs, TAAs, and NH_3_, as well as a reduction in the production of urea in the liver (*P* < 0.05) (Table 7). Similarly, relative to the pigs in the control group, pigs in the LP-DCA group showed reduced metabolism of Lys, Met, Thr, Trp, Arg, His, Ile, Leu, Phe, Val, Ser, Gly, Ala, Cys, Tyr, EAAs, NEAAs, TAAs, and NH_3_ and lower production of urea in the liver (*P* < 0.05). Pigs fed the LP-DCA diet showed reduced metabolism of Lys, Met, Thr, Arg, His, Ile, Leu, Phe, Val, Gly, Ala, EAAs, TAAs, and NH_3_ and reduced production of urea in the liver relative to those fed the LP diet (*P* < 0.05). The metabolism of Ser, Glu, Cys, Tyr, NEAAs in the liver of pigs fed the LP-DCA diet was higher than that of pigs fed the LP diet (*P* < 0.05).

**Table 7:** The effects of adding sodium dichloroacetate (DCA) to low-protein diets on the hepatic balance of AAs (mg/kg/h), NH_3_ (μmol/kg/h), and urea (mmol/kg/h) in pigs^*^

Items	Treatments			SEM	*P*
	Control	LP	LP-DCA		
Lys	4.90^a^	4.48^a^	2.94^b^	0.09	<.001
Met	1.97^a^	1.73^b^	1.17^c^	0.05	<.001
Thr	3.97^a^	3.59^a^	2.46^b^	0.15	<.001
Trp	2.07^a^	1.44^b^	1.33^b^	0.02	<.001
Arg	4.99^a^	4.81^a^	3.32^b^	0.08	<.001
His	3.19^a^	3.06^a^	1.75^b^	0.06	<.001
Ile	3.69^a^	3.11^b^	2.08^c^	0.06	<.001
Leu	3.27^a^	2.83^b^	1.99^c^	0.09	0.002
Phe	3.23^a^	3.04^a^	1.80^b^	0.08	<.001
Val	2.23^a^	2.04^a^	1.47^b^	0.08	<.001
Pro	3.50^a^	2.53^b^	2.68^b^	0.05	<.001
Asn	2.87^a^	1.71^b^	2.56^ab^	0.12	0.015
Ser	1.05^a^	−1.69^c^	0.77^b^	0.11	<.001
Gln	−14.9^a^	−20.8^b^	−16.1^a^	0.66	<.001
Gly	14.6^a^	13.2^b^	8.77^c^	0.18	<.001
Ala	16.5^a^	14.1^b^	10.6^c^	0.14	<.001
Cys	1.22^a^	−0.63^c^	0.18^b^	0.09	<.001
Tyr	2.23^a^	0.97^c^	1.05^b^	0.09	<.001
EAAs	33.5^a^	30.1^b^	20.3^c^	0.74	<.001
NEAAs	27.1^a^	9.41^c^	10.5^b^	1.01	<.001
TAAs	60.6^a^	39.5^b^	30.8^c^	1.71	<.001
NH_3_	69.8^a^	50.8^b^	40.4^c^	2.13	<.001
Urea	−2.06^a^	−1.50^b^	−1.13^c^	0.02	<.001

Data are presented as means (*n* = 5)

Abbreviations: *Control* 18.0% CP, *CP* Crude protein, *DM* Dry matter, *EAAs* Essential amino acids, *NEAAs* Non-essential amino acids, *LP* 13.5% CP, *LP-DCA* 13.5% CP + 100 mg DCA/kg DM, *SEM* Standard error of mean, *TAAs* Total amino acids

^*^A positive value means the amount of nitrogen-containing compound leaving the liver is less than that entering the liver; a negative value means the amount of nitrogen-containing compound leaving the liver is greater than that entering the liver

^a,b,c^Values within a row with different superscripts differ significantly (*P* < 0.05)

## Discussion

### Nitrogen balance

In this study, we found that the provision of a LP diet, regardless of whether it was supplemented with DCA, resulted in reduced nitrogen excretion by pigs. Specifically, compared with pigs in the control group, those in the LP and LP-DCA groups showed reductions in urinary nitrogen excretion by 23.5% and 44.7%, respectively, and reductions in fecal nitrogen of 35.0% and 38.6%, respectively. Although the reductions in the fecal nitrogen excretion of the two groups were comparable, the reduction in urinary nitrogen excretion in pigs fed a DCA-supplemented LP diet was considerably higher than that of pigs fed the unsupplemented LP diet. In addition, compared with pigs fed the control diet, we found that the pigs fed a LP diet without DCA supplementation showed reduced nitrogen retention. These observations are similar to the findings of our earlier study [17] and other studies [8, 51], which have reported that nitrogen retention was reduced when the amount of dietary CP was reduced by ≥3%. However, DCA supplementation compensated for the adverse effects of the LP diets on nitrogen retention in pigs, which is in accordance with our hypothesis that DCA may be a suitable dietary additive for reducing nitrogen excretion in pigs via an enhancement of nitrogen utilization efficiency. In this regard, we suspect that the mechanisms underlying the enhanced nitrogen utilization are related to the changes in nutrient metabolism in response to dietary DCA supplementation.

### Nutrient metabolism

In a previous study, we demonstrated that the provision of a LP diet supplemented with Lys, Met, Thr, and Trp reduced the supply of NEAAs to the portal vein and increased the rate of EAA metabolism in the liver of pigs [17]. NEAAs play important roles in the synthesis of numerous bioactive compounds and the normal growth and maintenance of animals [52, 53]. The daily body weight gain and feed efficiency of growing pigs are reduced when the dietary NEAA supply is insufficient [9]. Therefore, we assume that an insufficient supply of NEAAs is a major disadvantage of LP diets. In the present study, although the net amount of NEAAs and TAAs released into the portal vein of pigs fed a DCA-supplemented LP diet were still lower than those of pigs fed the control diet, addition of DCA was observed to reduce the metabolism of EAAs and TAAs in the liver. Eventually the differences in the fluxes of NEAAs, EAAs, and TAAs across the hepatic vein between the control group and the pigs that received a LP diet were abolished. In addition, compared with pigs in the control and LP groups, pigs in the LP-DCA group showed a reduced release of NH_3_ into the portal vein and reduced urea production in the liver. These results indicate that supplementing a LP diet with DCA can enhance nitrogen utilization efficiency in pigs by increasing and optimizing the supply of amino acids to the liver and extra-hepatic tissues, thereby reducing urea production in the liver and urinary nitrogen excretion. Notably, dietary DCA supplementation promoted a reduction in the catabolism of amino acids such as Glx (Glu + Gln) in the PDV, which are known to be major energy substrates in enterocytes and colonocytes and are beneficial for intestinal health [16, 54, 55]. Therefore, theoretically, dietary DCA supplementation may have some adverse effects on pigs, primarily on the intestines. We suggest that in future studies, more attention should be devoted to assessing the effects of DCA on intestinal health.

Recent evidence has indicated that gut microbiota in the intestines play an important role in dietary protein/amino acid metabolism. Fermentation of amino acids by gut bacteria produces metabolites that can affect host protein/amino acid uptake (transport) and metabolism, as well as influenc host cell physiology [56]. Moreover, bacteria can synthesize certain amino acids for host utilization [57]. Reducing the amount of dietary protein has been viewed as an alternative option that can be used to reduce excessive protein fermentation in the large intestine of pigs [58]. This reduction can inhibit the growth of potential pathogens, such as species of *Bacteroides* and *Clostridium*, and reduce amino acid deamination [59]. These studies indicate that the reductions in nitrogen excreted in the feces and urine of pigs fed LP diets with or without DCA supplementation may be related to a reduction in the amino acid deamination activities of intestinal microbiota. However, given that we did not investigate the effects of DCA on the intestinal microbiota in this study, we were unable to test this hypothesis.

We assume that enhanced amino acid utilization efficiency is closely related to changes in energy utilization efficiency. The LP diets formulated in the present study were designed to be isocaloric with the control diet, which we achieved by increasing the proportion of carbohydrates in the diet. Accordingly, the LP diets were characterized as being LP high-starch diets. The net energy provided by starch is considerably higher than that provided by the protein [60]. Feeding LP diets has been found to reduce the amount of energy required for deamination of excess amino acids, lower body protein turnover and heat production, and enhance energy utilization efficiency [61]. In the present study, we observed that there were no significant differences in the digestive energy digestibility and metabolic energy/digestive energy ratio of pigs provisioned with the control and LP diets, although the values recorded for pigs in the LP group tended to be higher, whereas those for pigs in the LP-DCA group were significantly higher. Accordingly, these results indicate that the energy utilization efficiency of pigs was enhanced by dietary DCA supplementation.

Of note in this regard is the fact that DCA is similar to acetate, which is known to serve as a source of energy in several cell types, including intestinal cells, and has profound effects on gut metabolism and health [62]. Although, to the best of our knowledge, there is no direct evidence to indicate that DCA can be used as an energy substrate or energy precursor similar to acetate, the dose of DCA used in the present study was very low. Thus, we could effectively ignore the putative role of DCA as an energy substrate/energy precursor, even though DCA is functionally similar to acetate.

### Possible effect mechanisms of DCA

We have previously shown that DCA supplementation can reduce the phosphorylation of PDK1 in porcine intestinal epithelial (IPEC-J2) cells and also promotes the phosphorylation of PDP1 and PDHA1 [28], which is consistent with the findings of other studies [32]. The phosphorylation of PDK and deactivation of PDH reduce carbohydrate flux [63, 64], whereas dephosphorylation and activation by PDP promote carbohydrate oxidation [65]. In addition, we have found that DCA supplementation increases cellular GDH1 activity and reduces GDH2 activity [28], the latter being responsible for catalyzing the conversion of glutamate to α-ketoglutarate (a substrate in the TCA cycle); the former plays the opposite role. Thus, supplementation of a LP diet would, in theory, promote glucose oxidation, while reducing the amount of amino acids entering the TCA cycle.

## Conclusions

In this study, we demonstrated that addition of DCA to LP diets can enhance the nitrogen utilization efficiency by increasing the amino acid supply from the intestines, reducing amino acid metabolism in the PDV and liver, and reducing the production of urea in the liver. Consequently, DCA supplementation increased the amino acid metabolic thrift and reduced nitrogen excretion without negatively affecting nitrogen retention in pigs fed a diet in which dietary CP was reduced by 4.5%. However, even though we have demonstrated the value of DCA as a dietary supplement, a considerable amount of additional research is necessary to determine the effects of DCA on growth, immunity, and meat quality before DCA can be widely used in pig diets.

## Data Availability

The data analyzed during the current study are available from the corresponding author on reasonable request.

## Abbreviations

AAs: Amino acids
CP: Crude protein
DCA: Sodium dichloroacetate
EAAs: Essential amino acids
GDH1: NADPH-dependent glutamate dehydrogenase
GDH2: NAD^+^-dependent glutamate dehydrogenase
GLUT: Glucose transporter
HAPF: Hepatic artery plasma flow
HVPF: Hepatic vein plasma flow
IR: Infusion rate
NEAAs: Non-essential amino acids
NH_3_: Ammonia
PAH: P-aminohippuric acid
PAH_hv_, PAH_ca_, and PAH_pv_: are the PAH concentrations in the hepatic vein, carotid artery, and portal vein, respectively
PDH: Pyruvate dehydrogenase
PDK: Pyruvate dehydrogenase kinase
PDP: Pyruvate dehydrogenase phosphatase
PVPF: Portal vein plasma flow
TAAs: Total amino acids
